# STATE POLICY AND POLITICAL LANDSCAPES: IMPLICATIONS FOR COGNITIVE AND FUNCTIONAL HEALTH

**DOI:** 10.1093/geroni/igae098.0692

**Published:** 2024-12-31

**Authors:** Marc Garcia, Blakelee Kemp, Wassim Tarraf

**Affiliations:** Syracuse University, Syracuse, New York, United States; University of Nebraska Lincoln, Lincoln, Nebraska, United States; Wayne State University, Detroit, Michigan, United States

## Abstract

There is considerable state-level variation in the percent of adults with cognitive and functional limitations. However, it is unclear how state policy contexts and changes in these contexts are associated with cognitive and functional health. We used BRFSS data (2015/16 and 2019/20) linked to the State Policy & Politics Data (SPPD) from the Center for Aging and Policy Studies (CAPS) at Syracuse University to examine how overall political ideology, Medicaid generosity, and three health behavior (Tobacco excise taxes, opioid laws, and clean-air indoor laws) policies and change in those policies over time (2000-2014) are linked to levels of perceived cognitive decline and functional limitations among individuals 45-years and older. We found considerable variability in both levels of perceived cognitive decline and functional limitations, as well as policy adoption and change in adoptions over time across states. Adjusting for individual-level sociodemographic indicators and change in state ideology/policy over time, higher levels of political liberalism, Medicaid generosity, Tobacco taxes, and more restrictive clean indoor air policies in 2014 were largely associated with lower levels of perceived cognitive decline and lower levels of functional limitations. However, greater change in political ideological and policy measures was inconsistently linked to higher prevalence of the considered impairment outcomes. As the U.S. ages and the landscape of state policies continues to diversify, policymakers are increasingly urged to conscientiously consider the cognitive and functional health implications associated with these varied policy environments.

